# Urban–rural disparities in the association between long-term exposure to high altitude and malnutrition among children under 5 years old: evidence from a cross-sectional study in Tibet

**DOI:** 10.1017/S1368980022001999

**Published:** 2023-04

**Authors:** Xianzhi Li, Yajie Li, Xiangyi Xing, Yu Liu, Zonglei Zhou, Shunjin Liu, Yunyun Tian, Qucuo Nima, Li Yin, Bin Yu

**Affiliations:** 1 Meteorological Medical Research Center, Panzhihua Central Hospital, Panzhihua, People’s Republic of China; 2 Clinical Research Center, Panzhihua Central Hospital, Panzhihua, People’s Republic of China; 3 Tibet Center for Disease Control and Prevention, Lhasa, People’s Republic of China; 4 Department of Pharmacy, Panzhihua Central Hospital, Panzhihua, People’s Republic of China; 5 Chongqing Center for Disease Control and Prevention, Chongqing, People’s Republic of China; 6 Department of Epidemiology, School of Public Health, Fudan University, People’s Republic of China; 7 Institute for Disaster Management and Reconstruction, Sichuan University – Hong Kong Polytechnic University, Chengdu, People’s Republic of China; 8 West China School of Public Health and West China Fourth Hospital, Sichuan University, Chengdu, People’s Republic of China

**Keywords:** Malnutrition, High altitude, Urban and rural area, Tibet

## Abstract

**Objective:**

To assess urban–rural disparities in the association between long-term exposure to high altitude and malnutrition among children under 5 years old.

**Design:**

A three-stage, stratified, cluster sampling was used to randomly select eligible individuals from July to October 2020. The data of participants, including demographic characteristics, altitude of residence, and nutritional status, were collected via questionnaire and physical examination.

**Setting:**

Tibet, China.

**Participants:**

Children under 5 years old in Tibet.

**Results:**

Totally, 1975 children under 5 years old were included in this study. We found that an additional 1000 m increase in altitude was associated with decreased Z-scores of height-for-age (*β* = –0·23, 95 % CI: –0·38, –0·08), Z-scores of weight-for-age (*β* = –0·24, 95 % CI: –0·39, –0·10). The OR for stunting and underweight were 2·03 (95 % CI: 1·51 to 2·73) and 2·04 (95 % CI: 1·38 to 3·02) per 1000 m increase in altitude, respectively; and OR increased rapidly at an altitude above 3500 m. The effects of long-term exposure to high altitudes on the prevalence of underweight in rural children were higher than that in urban children (*P* < 0·05).

**Conclusions:**

High-altitude exposure is tightly associated with malnutrition among children under 5 years old. Improving children’s nutrition is urgently needed in areas above 3500 m, especially in rural ones.

Malnutrition among children under 5 years old remains a major public health problem globally. A recent WHO report documented that more than 149·2 million children under 5 years old worldwide suffer from malnutrition, with the majority living in low- and middle-income countries in Africa or Asia^(1,2)^.

Current evidence suggests that in China, malnutrition in childhood has decreased in the past decade; for instance, the prevalence of stunting declined from 7·4 % in 2012 to 4·7 % in 2019^(2)^. However, a survey targeting poor rural counties showed that the total prevalence of malnutrition among children under 5 years old is 19·2 %^(3)^. It is well established that early-life malnutrition may lead to short-term and long-term adverse health outcomes (e.g. weak immunity, cognitive deficits and deaths^(1,4)^). It also contributes to a vicious cycle of health vulnerability: mothers who were malnourished in their early childhood are more likely to give birth to malnourished children^(5)^. These circumstances may make it difficult to reach the millennium development goal of reducing malnutrition and the UN’s Sustainable Development Goal to ‘improve nutrition’^(6)^.

The past decade has witnessed an increased number of researches to tackle malnutrition. Sociodemographic^(7,8)^, environmental factors^(9,10)^, socio-economic inequality^(7,10,11)^ and parasitic infectious diseases^(12)^ have been associated with malnutrition in children under 5 years old. It is widely acknowledged that environmental factors (e.g. geographical factors) are crucial to defining the human growth pattern characterised by anthropometric and function features (e.g. birth length and birth weight), and children located in different resident altitudes also exhibited significant heterogeneity in growth patterns^(9,13)^. Several studies conducted in Ethiopia^(14)^, Tibet^(15)^ and Ecuador^(16)^ revealed the negative or positive influence of geographical altitude on nutritional outcomes. A multi-centre study conducted in fifty-nine low- and middle-income countries suggested that residing at a higher altitude may be associated with child growth slowing even for children living in ideal home environments^(17)^. Indeed, geographical variations in the prevalence of severe malnutrition have been observed, which may reflect differences in the distribution, causes and effects of multiple risk factors for malnutrition^(6)^. Besides, regional averages can mask wide variations in country prevalence^(9)^, and the malnutrition issue in children living in high lands warrants significant attention.

Tibet is located on the Qinghai–Tibet Plateau at an average altitude above 4000 m. The harsh environment and urban–rural disparities account for the poor nutritional status of children^(15,18)^. A study in Tibet (in 1999) suggested that altitude is a crucial factor associated with malnutrition, and rural children exhibited a prevalence of 41·4 % and 24·7 % for stunting and underweight, higher than their urban counterparts^(15)^. The urban–rural gap was expected to decrease with the introduction of effective interventions and related policies (e.g. *China National Program for Child Development (2011–2020)*^(19)^) in the past decade. However, no follow-up studies have shed light on this issue in Tibet. Besides, previous studies have failed to consider urban–rural factor as potential effect modifier in the association between geographical altitude and malnutrition.

To fill these gaps, this study aimed to reveal the association between geographic altitudes and three anthropometric indicators of malnutrition (i.e. stunting, underweight and wasting) among children aged less than 5 years in Tibet, with emphasis on urban–rural disparities. Our study can provide substantial evidence regarding malnutrition issue in plateau areas.

## Methods

### Study population and sampling strategy

A three-stage, stratified, cluster sampling was used to randomly select eligible individuals from July to October 2020. Briefly, eight areas in Tibet (counties) were first selected proportional to the altitude and population size. Then, five towns or subdistricts were selected from each county as primary sampling units. In each of the forty primary sampling units, four villages or communities were randomly selected. Furthermore, both children aged 0–59 months and their parents living in the selected villages or communities were interviewed using structured questionnaires. Finally, a total of 2559 children were interviewed. Supplemental Figure S1 shows the sample size of county-level cities in Tibet.

**Fig. 1:**
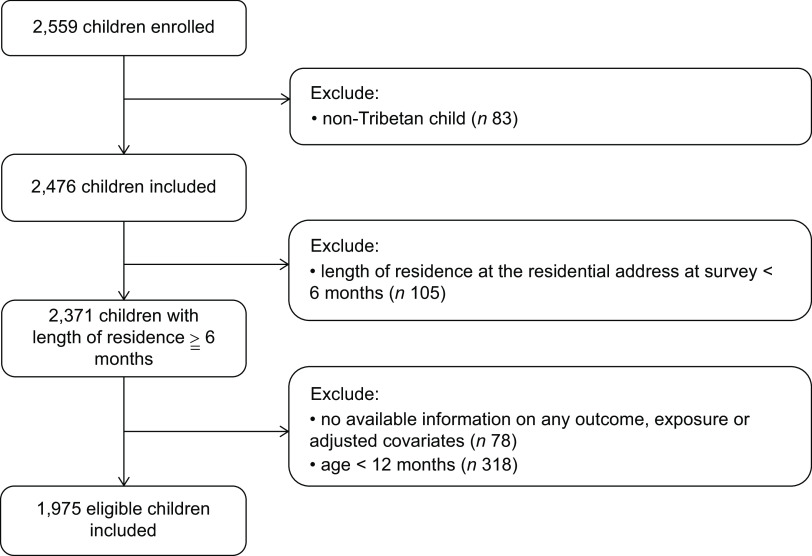
Flowchart of the study population

The exclusion criteria of this study were as follows: (1) non-Tibetan children; (2) children under the age of 12 months. To better control the influence of dietary factors and feeding practices on the associations between altitude and malnutrition, we exclude children aged under 12 months; (3) children with missing important variables; and (4) children who had lived at the survey site for less than 6 months. Ultimately, 1975 children were included in this study, with an enrolment rate of 77·2 % (Fig. 1).

### Data collection

Based on reports of previous studies and theoretical relevance, several covariates that may influence the associations between altitude and malnutrition indicators were included^(14,20–23)^. The definition and measurement of covariates are presented as follows:

(a) Age: measured in months.

(b) Gender: males and females.

(c) Asthma history, anaemia history and history of dental caries: assessed by whether the child had asthma, anaemia or dental caries prior to the data collection date and categorised into yes, no and not sure.

(d) Low birth weight: defined as birth weight less than 2500 g and dichotomised into yes and no.

(e) Optimal feeding practice scores: 24-h dietary recall method was used to collect information on dietary practice to analyse dietary diversity and meal frequency. According to the *WHO Infant and Young Children Feeding Practice Guidelines*,^(24)^ the minimum dietary diversity was met if a child took four or more of the seven food groups (including (i) grains, roots and tubers, (ii) legumes and nuts, (iii) dairy products, (iv) flesh foods, (v) eggs, (vi) vitamin A-rich fruits and vegetables, (vii) other fruits and vegetables) on the previous day; the minimum meal frequency was met if a child ate meal more than three times on the previous day. Besides, data on breast-feeding and complementary food feeding were also collected, including (i) whether the child was breastfed within 1 h of birth, (ii) whether the child was fed exclusively with breast milk until 6 months of age, (iii) whether the child was given complementary foods between 6 and 8 months of age, (iv) whether the child was consistently breastfed until 12 months of age, and (v) whether the child was fed an Fe-rich food or Fe-fortified food on the previous day. For the above-mentioned seven items, we assigned each item a score of 0 or 1. Then, we summed the scores for the seven items to obtain an optimal feeding practice score. Optimal feeding practice scores were divided into low and high scores based on the median feeding practice scores (median = 3).

(f) Maternal educational level: assessed by the highest educational level completed by the mother of the child and categorised into illiterate, primary school and junior high school or above.

(g) Maternal height: measured objectively in cm and categorised into three groups: < 160·0cm, 160·0–169·9 cm and ≥ 170·0 cm. The maternal height categories were adapted from several earlier studies^(25–27)^.

(h) Maternal weight: measured objectively in kg and categorised into three groups: < 50·0 kg, 50·0∼59·9 kg and ≥ 60·0 kg. The weight categories were adapted from previous study^(14)^.

(i) Mother suffering from anaemia during pregnancy: assessed by whether the mother suffered from anaemia during pregnancy and dichotomised into yes and no.

(j) Antenatal visits: assessed by the number of antenatal visits attended by the mother during the pregnancy and categorised into three groups: none, < 8 times and ≥ 8 times.

(k) Wealth status: assessed by yearly family income and categorised into five groups based on the quintile of Chinese household annual income from China Statistical Yearbook (2019): poorest (< 12 000 Chinese Yuan (CNY)), poorer (12 000–19 999 CNY), middle (20 000–39 999 CNY), richer (40 000–59 999 CNY) and richest (> 60 000 CNY).

(l) Drinking water source: assessed by the type of water source used by the household and dichotomised into improved and unimproved. According to WHO guideline, improved water sources referred to piped water and protected wells, and unimproved water sources referred to springs, lakes, ponds, unprotected wells, rivers and dams.

(m) Residence: assessed by the place of residence and dichotomised into rural areas and urban areas based on the urban–rural classification code formulated by the National Bureau of Statistics of the People’s Republic of China (2020).

### Exposure assessment

The primary exposure in our study was the altitude of participants’ residence. Global Positioning System (GPS) was used to measure and record the altitude and geographical location of survey spots. Supplemental Figure S1 shows the average altitude of prefecture-level cities in Tibet.

### Outcome assessment

Stunting, underweight and wasting were the primary outcomes of our study. Underweight can be attributed to acute and chronic malnutrition, while stunting and wasting are related to chronic and acute malnutrition, respectively^(28)^. The length was measured in a recumbent position three times for children younger than 2 years of age, while height was measured in a standing position three times for children older than 2 years of age (for convenience, both length and height were referred to as height below). The weight was measured three times, with participants wearing fewer clothes and bare feet. The above measurements were averaged to calculate the height and weight, respectively. According to the WHO’s 2006 Child Growth Standard^(29)^, Z-scores were defined as dividing the difference between the observed value and the mean value of the reference population by the sd of the reference population. Stunting, underweight and wasting in children were defined as Z-scores < –2 for height-for-age (HFA), weight-for-age (WFA) and weight-for-height (WFH), respectively.

### Statistical analysis

**Fig. 2:**
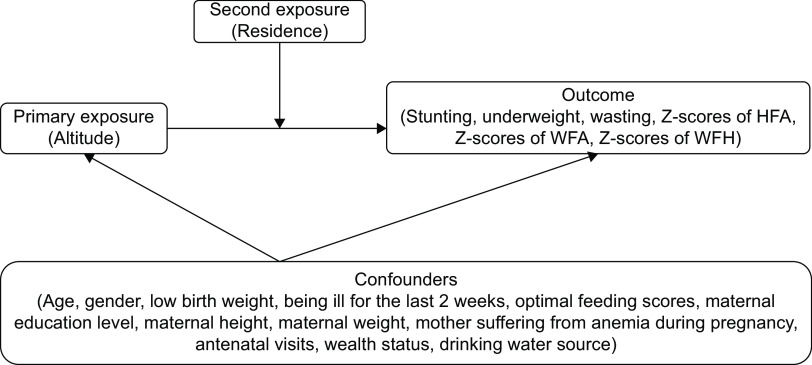
A brief diagram of relationships among the outcomes, exposures and confounders

We preliminary show relationships among the outcomes, exposures and confounders by a brief diagram (Fig. 2). Furthermore, structural equation model (sem) causal diagrams were used to elaborate on relationships among the primary outcomes, exposures and confounders (see online supplementary material, Supplemental Figure S2–S4).

We measure the child’s nutrition status using both continuous and dichotomous variables. We evaluated the long-term effects between altitude and continuous measurement for nutrition using multivariable linear regression models and reported the effect estimates *β* and 95 % CI. We evaluated long-term effects between altitude and dichotomous outcome using multivariable logistic regression models and reported the OR and 95 % CI. Based on the previous literature on malnutrition and altitude among children, fully adjusted models included the following covariates: age, gender, low birth weight, being ill during the last 2 weeks, optimal feeding scores, maternal educational level, maternal height, maternal weight, mother suffering from anaemia during pregnancy, antenatal visits, wealth status and drinking water source. All associations were reported per 1000 m increase of altitude.

We performed analyses stratified by the participants’ residence (rural areas *v*. urban areas), gender (male *v*. female), age (12–35 months *v*. 36–60 months), wealth status (low *v*. high income), drinking water source (improved *v*. unimproved) and optimal feeding scores (high scores *v*. low scores) to examine whether the associations were consistent among different subpopulations. The Z test was used to examine the differences between subgroups.

We also performed sensitivity analyses by two strategies. We adjusted for three pre-existing diseases including asthma, anaemia and dental caries to minimise the influence of the above three diseases. Besides, the linear relationship between altitude and six malnutrition indicators was explored using penalised spline models with four knots at the 5th, 35th, 65th and 95th centiles, respectively. We specified the HR or *β*-value as 1 at 3590 m (median of altitude). All statistical analyses were performed using R 4.1.0, and statistical significance was declared if *P* < 0·05.

## Results

### Demographic characteristics

Table 1 reports the characteristics of the study population, which included 1975 children aged between 12 and 59 months. Among these children, 1647 and 328 lived in rural and urban regions, respectively. Compared with rural children, urban children were more likely to be older, suffering from dental caries, breastfed exclusively until 6 months and consistently breastfed until 12 months, with a higher dietary diversity, better wealth status and cleaner drinking water source. The mothers of urban children tended to have better education levels, higher height, heavier weight and more antenatal visits than their counterparts. The children in rural areas were given complementary foods between 6 and 8 months of age, with a greater meal frequency.

**Table 1:** Basic characteristics of study participants

Characteristics	Total (*n* 1975)		Urban area (*n* 328)		Rural area (*n* 1647)		*P*-value
	*n*	%	*n*	%	*n*	%	
Gender							
Female	937	47·4	161	49·1	776	47·1	0·514
Male	1038	52·6	167	50·9	871	52·9	
Age, months							
12–35	964	48·8	17	5·2	947	57·5	< 0·001
36–59	1011	51·2	311	94·8	700	42·5	
Low birth weight							
Yes	123	6·2	14	4·3	109	6·6	0·108
No	1852	93·8	314	95·7	1538	93·4	
Asthma history							
No	1797	91·0	306	93·3	1491	90·5	0·067
Yes	25	1·3	6	1·8	19	1·2	
Not sure	153	7·7	16	4·9	137	8·3	
Anaemia history							
No	1713	86·7	276	84·1	1437	87·2	0·154
Yes	262	13·3	52	15·9	210	12·8	
Suffering from dental caries							
No	1771	89·7	289	88·1	1482	90·0	0·032
Yes	92	4·7	24	7·3	68	4·1	
Not sure	112	5·7	15	4·6	97	5·9	
Being ill during the last 2 weeks							
No	1713	86·7	276	84·1	1437	87·2	0·154
Yes	262	13·3	52	15·9	210	12·8	
Child breastfed within 1 h after delivery							
No	703	35·6	14	4·3	689	41·8	< 0·001
Yes	183	9·3	2	0·6	181	11·0	
Not sure	1089	55·1	312	95·1	777	47·2	
Exclusive breast-feeding until 6 months							
Yes	1177	59·6	229	69·8	948	57·6	< 0·001
No	798	40·4	99	30·2	699	42·4	
Child given complementary foods between 6 and 8 months of age							
Yes	556	28·2	76	23·2	480	29·1	0·017
No	917	46·4	175	53·4	742	45·1	
Not sure	502	25·4	77	23·4	425	25·8	
Child consistently breastfed until 12 months							
Yes	852	43·1	207	63·1	645	39·2	< 0·001
No	1123	56·9	121	36·9	1002	60·8	
Dietary diversity							
< 4	418	21·2	18	5·5	400	24·3	< 0·001
≥ 4	1557	78·8	310	94·5	1247	75·7	
Meal frequency, times							
< 3	40	2·0	19	5·8	21	1·3	< 0·001
≥ 3	1935	98·0	309	94·2	1626	98·7	
Maternal educational level							
Illiteracy	1016	51·5	19	5·8	997	60·5	< 0·001
Primary school	340	17·2	17	5·2	323	19·6	
Junior high school or above	619	31·3	292	89·0	327	19·9	
Maternal height, cm							
160·0	576	29·2	61	18·6	515	31·3	< 0·001
160·0–169·9	1299	65·8	244	74·4	1055	64·0	
≥ 170·0	100	5·0	23	7·0	77	4·7	
Maternal weight, kg							
< 50·0	399	20·2	61	18·6	338	20·5	< 0·001
50·0–59·9	1186	60·1	173	52·7	1013	61·5	
≥ 60·0	390	19·7	94	28·7	296	18·0	
Mother suffering from anaemia during pregnancy							
No	1501	76·0	264	80·5	1237	75·1	< 0·001
Yes	186	9·4	38	11·6	148	9·0	
Not sure	288	14·6	26	7·9	262	15·9	
Antenatal visits, times							
< 8	1370	69·4	131	39·9	1239	75·2	< 0·001
≥ 8	343	17·4	188	57·3	155	9·4	
None	262	13·2	9	2·7	253	15·4	
Wealth status							
Poorest	448	22·7	45	13·7	403	24·5	< 0·001
Poorer	848	42·9	56	17·1	792	48·1	
Middle	350	17·7	31	9·5	319	19·4	
Richer	264	13·4	141	43·0	123	7·5	
Richest	65	3·3	55	16·7	10	0·6	
Drinking water source							
Improved	1443	73·1	314	95·7	1129	68·5	< 0·001
Unimproved	532	26·9	14	4·3	518	31·5	

Table 2 shows the average altitude at which the participants lived and malnutrition indicators. The average altitude in rural and urban areas was 3550 m (interquartile range, 3250 m to 4520 m) and 3660 m (interquartile range, 3550 m to 4500 m), respectively; and there was no statistical difference in altitude between rural and urban areas; Supplemental Fig. S5 showed the residential altitude of participants in rural and urban areas in more detail through the box chart. The Z-scores of HFA and WFA among urban children were higher than among rural children, but there was no statistical difference in Z-scores of WFH between the two groups. The prevalence rates of stunting and underweight in urban areas were lower than in rural areas, but there was no statistical difference in the prevalence of wasting (Table 2).

**Table 2:** Descriptive statistics of the average height of altitude and nutritional indicators of children by residence

Variables	Total		Urban area		Rural area		*P*-value
	Median	25 %, 75 %	Median	25 %, 75 %	Median	25 %, 75 %	
Altitude (m)	3590	3250, 4520	3660	3550, 4500	3550	3250, 4520	0·236
Z-scores of HFA	−0·76	–1·67,0·11	0·15	–0·61, 0·97	−0·92	–1·81, –0·13	< 0·001
Z-scores of WFA	−0·49	–1·21,0·33	−0·13	–0·62, 1·02	−0·58	–1·30, 0·16	< 0·001
Z-scores of WFH	0·07	–0·68,0·88	0·06	–0·56, 0·77	0·07	–0·69, 0·89	0·849
	*n*	%	*n*	%	*n*	%	*P*-value
Stunting*							< 0·001
Yes	363	18·4	15	4·6	348	21·1	
No	1612	81·6	313	95·4	1299	78·9	
Underweight†							< 0·001
Yes	161	8·2	10	3·0	151	9·2	
No	1814	91·8	318	97·0	1496	90·8	
Wasting‡							0·163
Yes	89	4·5	10	3·0	79	4·8	
No	1886	95·5	318	97·0	1568	95·2	

HFA, height-for-age; WFA, weight-for-age; WFH, weight-for-height.

* Stunting: Z-scores of HFA < –2.

† Underweight: Z-scores of WFA < –2.

‡ Underweight: Z-scores of WFA < –2.

### Associations between altitude exposure and malnutrition indicators

Table 3 shows the OR, *β* and 95 % CI for the association between the malnutrition indicators and a 1000 m increase in altitude exposure. Statistically significant changes in the Z-scores of HFA, WFA, and WFH, and stunting, underweight and wasting were observed per 1000 m increments in altitude in the crude model. Briefly, each 1000 m increase in altitude was associated with decreased Z-scores of HFA (*β* = –0·43, 95 % CI: –0·54, –0·33), WFA (*β* = –0·39, 95 % CI: –0·49, –0·29), WFH (*β* = –0·21, 95 % CI: –0·33, –0·09), and OR for stunting (2·34, 95 % CI: 1·95, 2·80), underweight (2·12, 95 % CI: 1·65, 2·73) and wasting (1·66, 95 % CI: 1·20, 2·29).

**Table 3:** Association of risk of nutritional indicators of children per 1000 m increase in altitude

Outcome	Altitude					
	Crude model†		Adjusted model 1‡		Adjusted model 2§	
	*β*	95 % CI	*β*	95 % CI	*β*	95 % CI
Z-scores of HFA	−0·43	–0·54, –0·33***	−0·42	–0·53, –0·32***	−0·23	–0·38, –0·08**
Z-scores of WFA	−0·39	–0·49, –0·29***	−0·38	–0·48, –0·28***	−0·24	–0·39, –0·10**
Z-scores of WFH	−0·21	–0·33, –0·09***	−0·20	–0·33, –0·08**	−0·18	–0·36, 0·00

	OR	95 % CI	OR	95 % CI	OR	95 % CI
Stunting||	2·34	1·95, 2·80***	2·32	1·92, 2·81***	2·03	1·51, 2·73***
Underweight¶	2·12	1·65, 2·73***	2·01	1·55, 2·62***	2·04	1·38, 3·02***
Wasting††	1·66	1·20, 2·29**	1·58	1·13, 2·21**	1·19	0·73, 1·93

HFA, height for age; WFA, weight-for-age; WFH, weight-for-height.

** *P*-value is between 0·001 and 0·01.

*** *P*-value < 0·001.

† Crude model: no adjustment.

‡ Adjusted model 1: adjusted for age, gender, low birth weight and being ill for the last 2 weeks.

§ Adjusted model 2: adjusted for age, gender, low birth weight, being ill for the last 2 weeks, optimal feeding scores, residence, maternal educational level, maternal height, maternal weight, mother suffering from anaemia during pregnancy, antenatal visits, wealth status and drinking water source.

|| Stunting: Z-scores of HFA < –2.

¶ Underweight: Z-scores of WFA < –2.

†† Underweight: Z-scores of WFA < –2.

After adjusting for confounders (adjusted model 1), the effect estimates between malnutrition indicators and altitude exposure were slightly lower. The results remained consistent except for wasting after adjusting for maternal and family factors (adjusted model 2).

### Stratified analysis

**Fig. 3:**
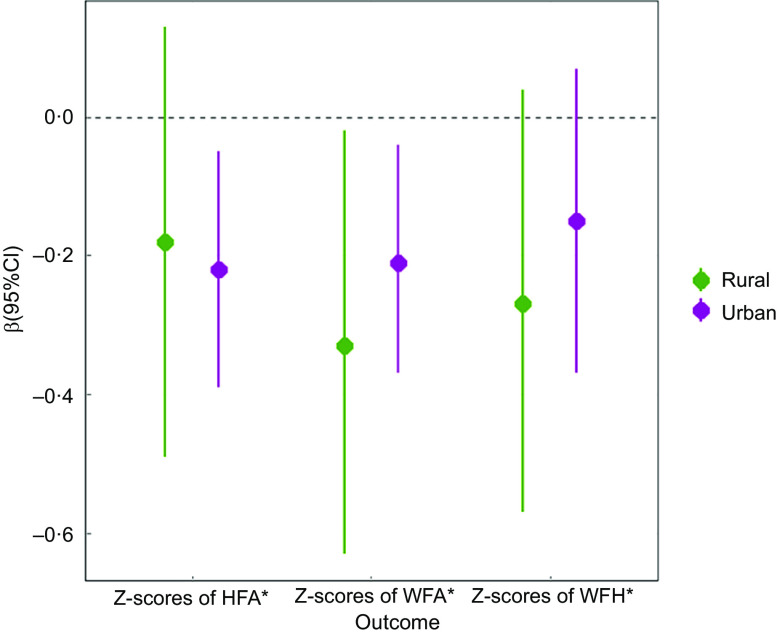
Associations between altitude and continuous malnutrition indicators of children stratified by residence. Notes: HFA, height-for-age; WFA, weight-for-age; WFH, weight-for-height. The adjusted models were adjusted for age, gender, low birth weight, being ill for the last 2 weeks, optimal feeding scores, maternal educational level, maternal height, maternal weight, mother suffering from anaemia during pregnancy, antenatal visits, wealth status and drinking water source. *P-value for difference: Z test was used to test for statistically significant difference in *β* estimates across categories within subgroups. For example, in rural area *v*. urban area, we calculated: *Z* = 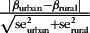

**Fig. 4:**
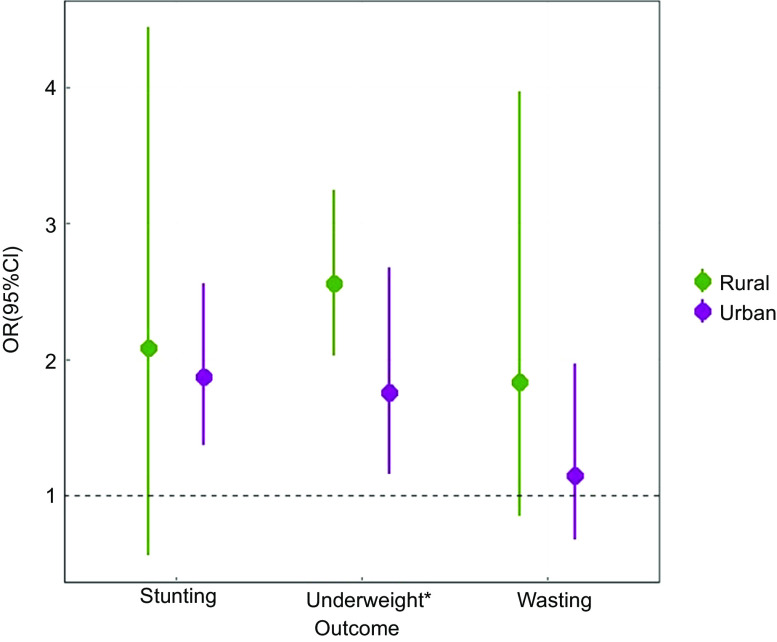
Associations between altitude and categorical malnutrition indicators of children stratified by residence. *Notes:* HFA, height-for-age; WFA, weight-for-age; WFH, weight-for-height. Stunting: Z-scores of HFA < –2. Underweight: Z-scores of WFA < –2. Underweight: Z-scores of WFA < –2. The adjusted models were adjusted for age, gender, low birth weight, being ill for the last 2 weeks, optimal feeding scores, maternal educational level, maternal height, maternal weight, mother suffering from anaemia during pregnancy, antenatal visits, wealth status and drinking water source. *P-value for difference: Z test was used to test for statistically significant difference in OR estimates across categories within subgroups. For example, in rural area *v*. urban area, we calculated: *Z* = 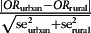

Figure 3 and 4 depict the results of the stratified residence analyses for the relationship of altitude with malnutrition indicators. For Z-scores of WFA, WFH and underweight, the associations were stronger in rural areas than in urban areas. For example, the association between underweight and per 1000 m increase in altitude was significantly higher among rural residents than urban residents. As for Z-scores of HFA, the associations were stronger in urban areas than in rural areas. Finally, the differences in effect estimations for stunting and wasting between urban and rural areas were not statistically significant.

Supplemental Figures S6 to S11 show the results of the stratification analysis except residence. In brief, altitude exerted a more significant effect on the Z-scores of HFA, WFA and WFH in individuals who were male, had low income and had unimproved drinking water sources. A greater effect of altitude on stunting was observed among younger children who had unimproved drinking water sources. As for underweight, the effect among children who had an unimproved drinking water source was greater.

### Sensitivity analysis

Our results on the effect of altitude on malnutrition remained consistent after adjusting for three pre-existing diseases including asthma, anaemia and dental caries (see online supplementary material, Supplemental Table S1). Additionally, in Supplemental Fig. S12, we used restricted cubic splines to flexibly model and visualise the relationships between altitude exposure and malnutrition measures, and non-linear relationships were found. Interestingly, the exposure and response curves between altitude and stunting or underweight showed a substantial increase of the risk at an altitude greater than 3500 m, and the associations were not statistically significant at an altitude below 3500 m. Nonetheless, for the exposure and response curve between altitude and wasting, the above phenomenon was not found.

## Discussion

In the present study, strong positive associations were found between residential altitude and stunting and underweight. The association between residential altitude and underweight prevalence in rural children was stronger than in urban children. Accordingly, we provide hitherto undocumented evidence on the urban–rural differences in the association of residential altitude with malnutrition in high-altitude areas in China.

Current evidence suggests that children living in high-altitude areas are at increased risk of poor growth and chronic malnutrition^(30)^. Our study found a positive association between residential altitudes and stunting or underweight, consistent with the previous studies conducted in highlands countries. In Argentina, underweight, stunting and wasting risks were higher at a higher altitude^(31)^. In Ethiopia, the odds of stunting were 1·41 times higher at altitude ≥ 2500 m compared with altitude < 1000 m^(32)^. In Tanzania, the prevalence of underweight was 1·91 times higher in highlands than in lowlands^(33)^. However, several studies found no significant association between altitude and malnutrition^(18,34)^. It should be borne in mind that the residential altitude of survey sites might be an important factor affecting the relationship between altitude and malnutrition, accounting for the above findings. A non-linear relationship in the odds of stunting and underweight with increasing altitudes was presented in our study, and the associations were not statistically significant at altitudes below 3500 m, suggesting the effect of residential altitude on children’s growth at high altitudes.

Our study found a statistically significant association between altitude and stunting or underweight, but not wasting. A growing body of evidence from recently published studies suggests that living at a high residential altitude significantly impacts linear growth (height) than body mass^(15,35)^. A close association between stunting and altitude implied that height was more susceptible to high altitude. Furthermore, the relationship between underweight and altitude might be attributed to the contribution of height to weight of children to a certain extent^(15)^. Moreover, the socio-economic status and genetic background could cause this difference, but the possibility that altitude affects linear growth should not be ruled out^(36)^. Moreover, the association between residential altitude and wasting was non-significant, given that wasting indicates acute malnutrition^(28)^. Indeed, child malnutrition results from long-term effects of environmental factors, especially residential altitude.

Despite evidence that the association between residential altitude and stunting or underweight is consistent, the underlying mechanism remains unclear. Research suggests that the growth pattern of children is determined by environmental factors, at least for children under 5 years old, although genes limit the potential for growth and development^(37)^. Overwhelming evidence substantiates that residential altitude directly impacts children’s growth through environmental stressors, including hypobaric hypoxia, high cosmic radiation, low temperatures and relative humidity^(37–39)^. In addition, a high residential altitude may indirectly lead to delayed growth in children. Previous studies have reported associations between high residential altitudes and low birth weight^(40,41)^, especially intra-uterine growth retardation-related low birth weight^(42)^, which may have subsequently resulted in delayed growth^(42)^. In some instances, limited nutritional resources in high-altitude areas are conducive for malnutrition in children^(43)^. However, these hypotheses have not been well explored, and there is no conclusive evidence to show the exact mechanism by which altitude negatively influences growth. Future research should comprehensively explore the direct and indirect effects of altitude on children’s malnutrition, to formulate targeted intervention measures for children’s nutrition improvement in high-altitude areas.

Moreover, we found that the association of residential altitude with underweight in urban children was stronger than in rural children. Limited research has been performed on the urban–rural differences in the association between residential altitude and malnutrition. In a cross-sectional study, urban native children exhibited better nutritional status in all age groups than rural children living at the same altitude^(44)^. S Dang et al.^(15)^ reported a dose–response relationship in the increased prevalence of stunting and underweight with increasing altitudes in rural areas, not in urban areas, which may be attributed to the prevalence of underweight among highlanders related to their low socio-economic status, including poor access to nutrients^(38)^. Ample evidence indicates that the difference in socio-economic status between urban and rural areas is bigger than between agricultural and pastoral areas in Tibet^(15)^. Furthermore, rural children may be more vulnerable to higher residential altitude due to the harsh living environments, limited nutritional resources, low-income family nurturing care and insufficient healthcare services^(45,46)^. Finally, compared with urban children, rural children are more likely to develop health risk behaviours, such as the absence of handwashing and tooth brushing, and excessive screen time^(47,48)^. Therefore, children in rural areas are more vulnerable to pneumonia and diarrhoea, leading to malnutrition.

The stratification analysis showed that children with unimproved drinking water sources at high residential altitudes were at higher risk of stunting and underweight, affected by, while younger children were at higher risk of stunting related to their greater vulnerability to environmental factors^(37)^. Children with unimproved drinking water source are prone to diarrhoea, which is associated with stunting and underweight^(49,50)^.

Our study has several strengths. First, we comprehensively evaluated the malnutrition status of Tibetan children by calculating six malnutrition indicators, including Z-scores of HFA, WFA, WFH, and OR for stunting, underweight, and wasting. Besides, we explored the modification effect of residence on the association between altitude and malnutrition by stratified analyses. Nevertheless, our study also had certain limitations. First, the residence altitude of most participants was above 3000 m, and hence, the conclusion of this study was mainly applicable to high-altitude areas. Moreover, it was challenging to establish causal relationships between different variables, since our study outcomes were collected at only one time point. Finally, it was impossible to adjust for all potential confounding factors. For example, the optimal feeding scores were calculated by breast-feeding and frequency of food consumption in the past, and no consideration was given to food or breast milk intake.

## Conclusion

Long-term exposure to high altitude is associated with increased risks of stunting and underweight among Tibetan children, and the risks increase rapidly at altitude above 3500 m. We found that the association of residential altitude on the underweight was more significant in rural areas than in urban areas. Accordingly, improving children nutrition is urgently needed in areas above 3500 metres, especially in rural ones.

## Supplementary material

For supplementary material accompanying this paper visit https://doi.org/10.1017/S1368980022001999. click here to view supplementary material
