# Supplementary material for: Urban–rural disparities in the association between long-term exposure to high altitude and malnutrition among children under 5 years old: evidence from a cross-sectional study in Tibet
Source: Public Health Nutr. 2022 Sep 13;26(4):844–53. doi: 10.1017/S1368980022001999 (PMC10131156; doi:10.1017/S1368980022001999)
Supplement: Supplementary file 1 [file S1368980022001999sup001.docx]

**SUPPLEMENTARY MATERIAL**

**Figure S1:** Maps of residential altitude of participants and sample sizes.

**Figure S2:** Structural equation model (SEM) causal diagram among the stunting, exposures, and confounders.

**Figure S3:** Structural equation model (SEM) causal diagram among the underweight, exposures, and confounders.

**Figure S4:** Structural equation model (SEM) causal diagram among the wasting, exposures, and confounders.

**Figure S5:** Residential altitude of participants in rural and urban areas.

**Figure S6:** Association between altitude and Z-scores of HFA stratified by potential modifiers.

**Figure S7:** Association between altitude and Z-scores of WFA stratified by potential modifiers.

**Figure S8:** Association between altitude and Z-scores of WFH stratified by potential modifiers.

**Figure S9:** Association between altitude and stunting stratified by potential modifiers.

**Figure S10:** Association between altitude and underweight stratified by potential modifiers.

**Figure S11:** Association between altitude and wasting stratified by potential modifiers.

**Table S1:** Association of malnutrition indicators of children per 1,000 m increments in altitude after adjusting for three preexisting diseases including asthma, anemia, and dental caries.

**Figure S12:** Curve associations between malnutrition indicators and altitude exposure.

**
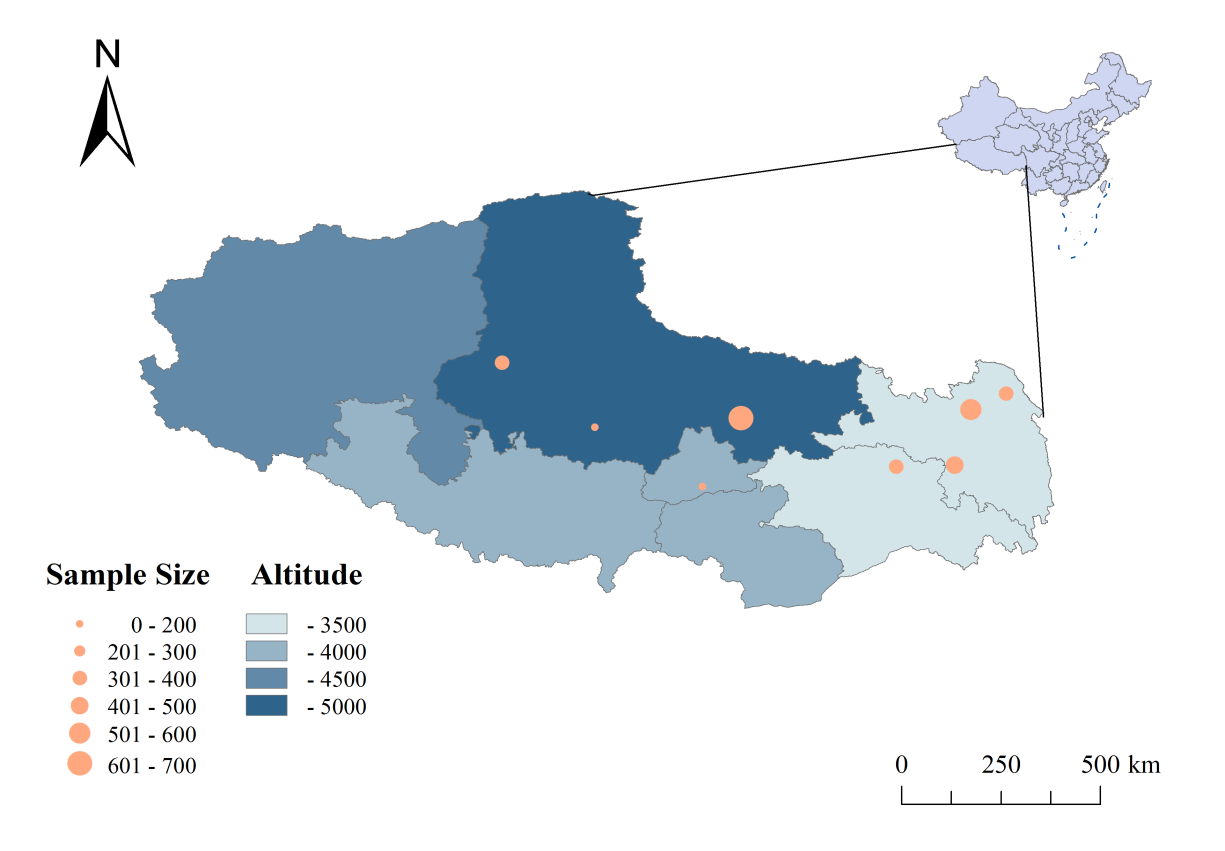
**

**Figure S1:** Maps of residential altitude of participants and sample sizes.


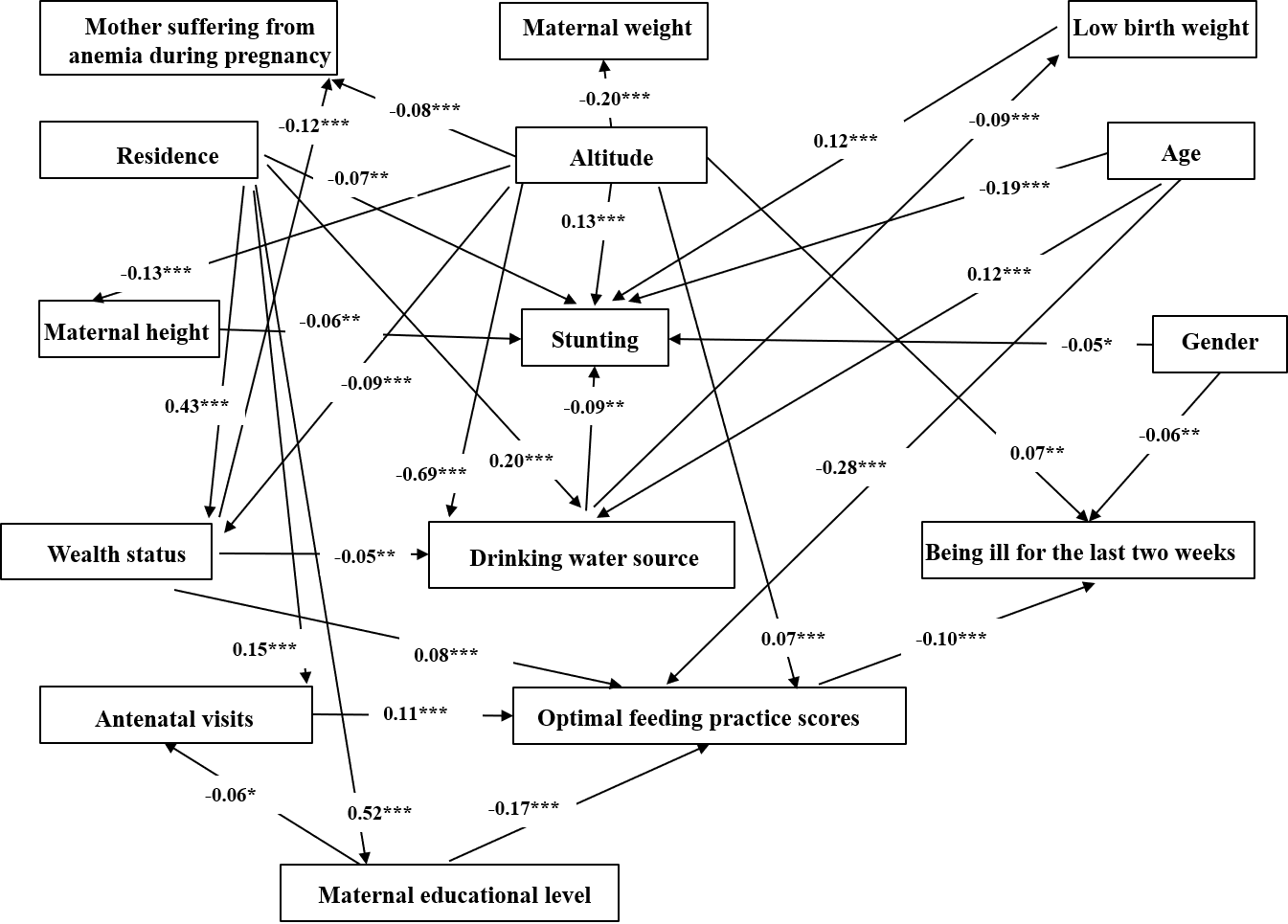
**Figure S2:** Structural equation model (SEM) causal diagram among the stunting, exposures, and confounders.

*Notes:* Values indicate standardized coefficients, * *P*<0.05, ** *P*<0.01, ****P*<0.001.


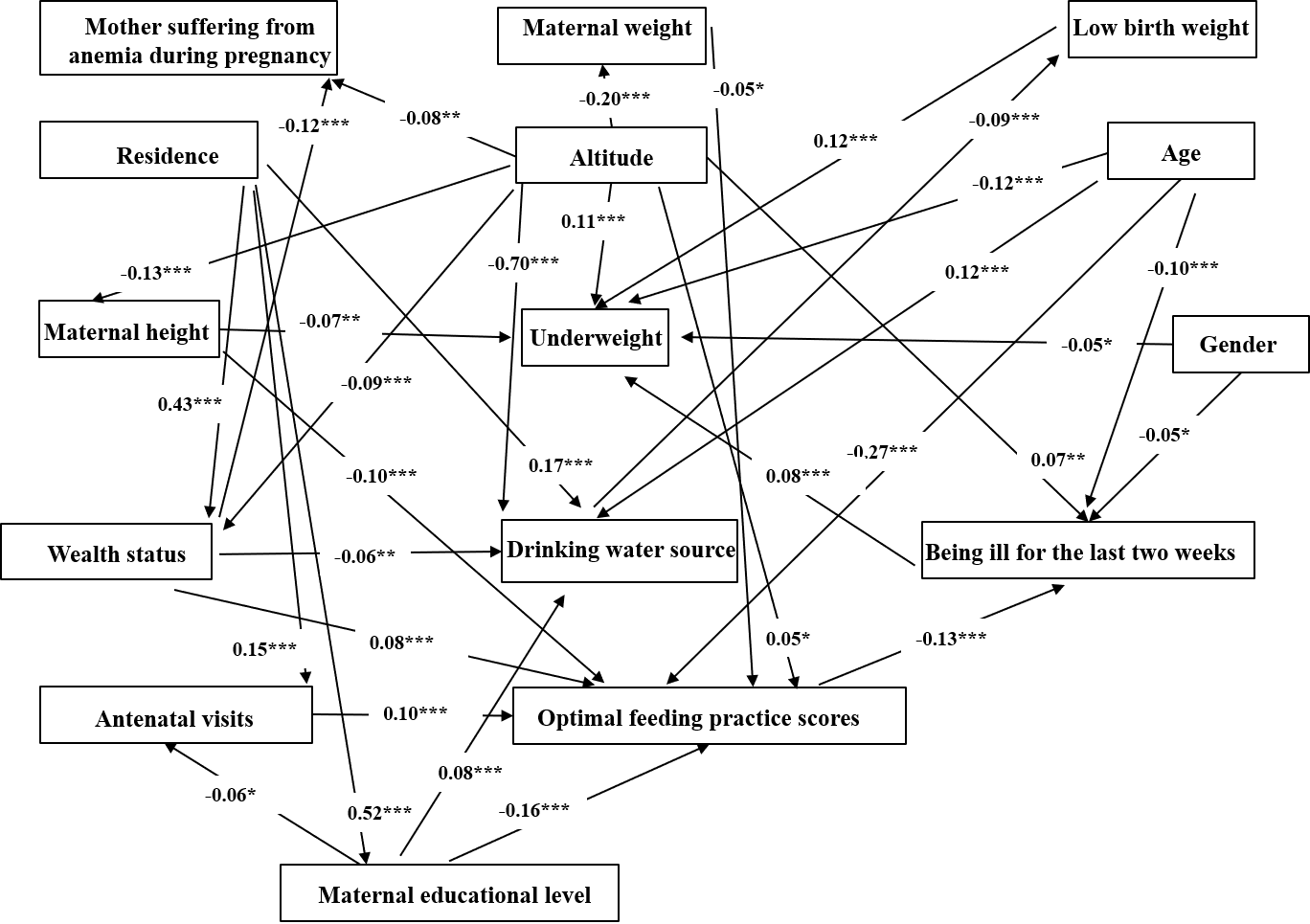
**Figure S3:** Structural equation model (SEM) causal diagram among the underweight, exposures, and confounders.

*Notes:* Values indicate standardized coefficients, * P<0.05, ** P<0.01, ***P<0.001.


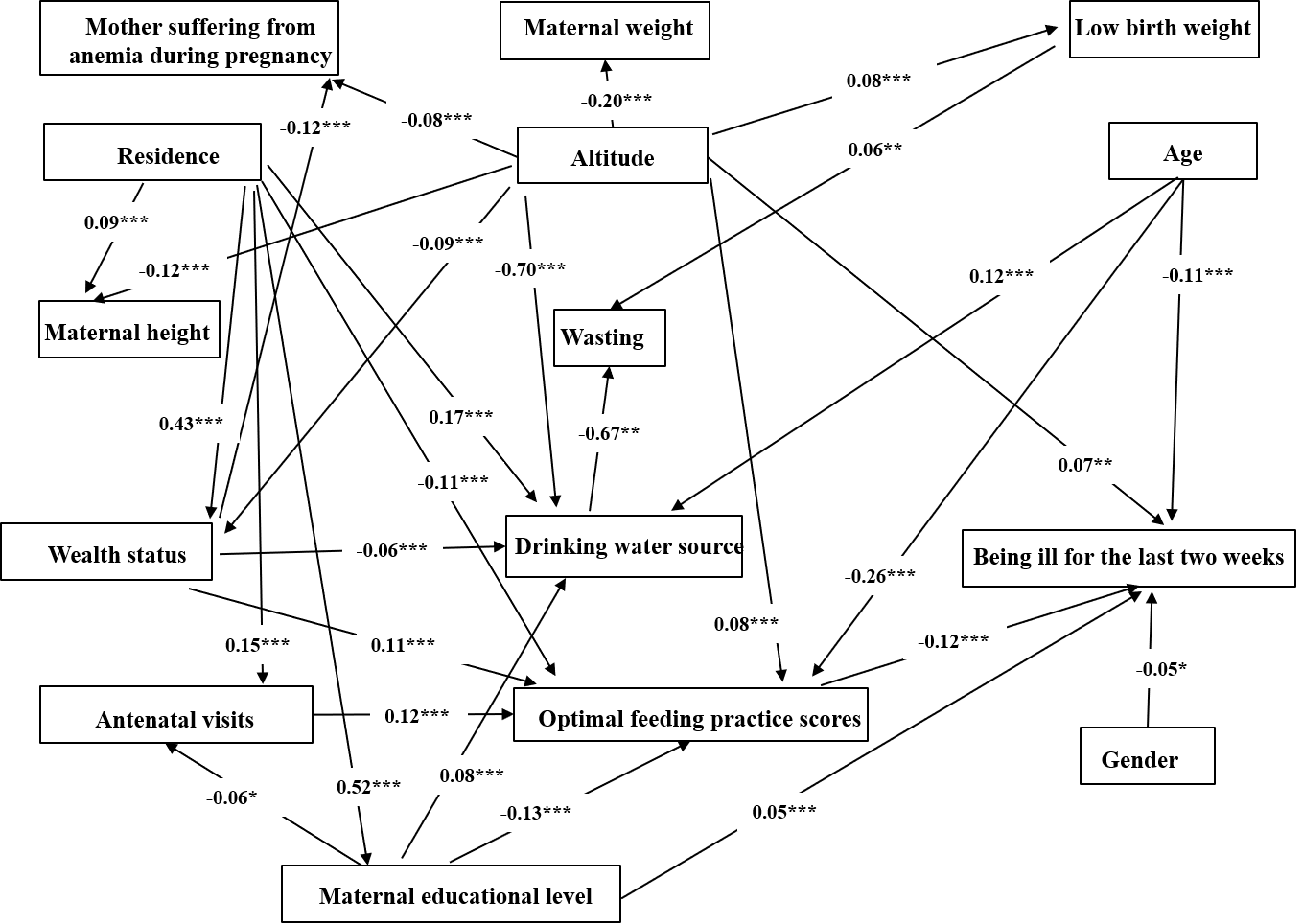
**Figure S4:** Structural equation model (SEM) causal diagram among the wasting, exposures, and confounders

*Notes:* Values indicate standardized coefficients, * *P*<0.05, ** *P*<0.01, ****P*<0.001.


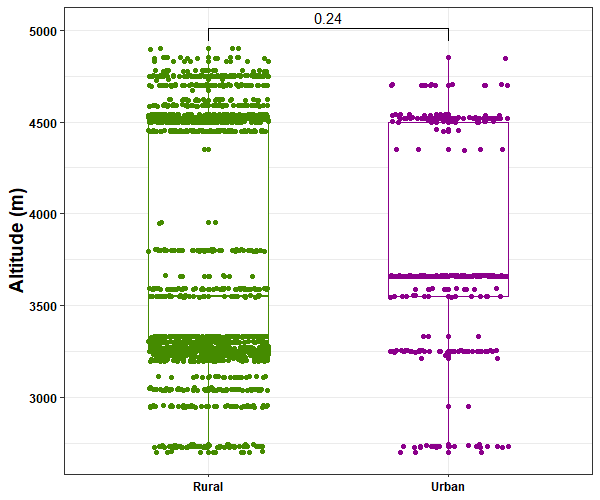
**Figure S5:** Residential altitude of participants in rural and urban areas.

*Notes:* Value (0.24) represents the *P* value of the comparison between the two groups.

**
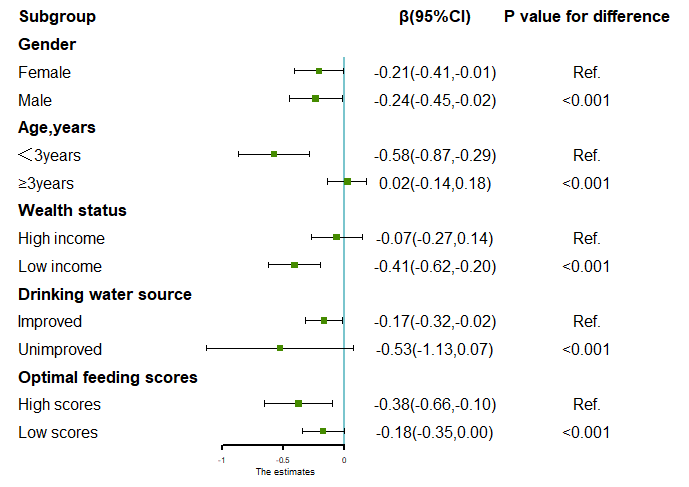
Figure S6 Association between altitude and Z-scores of HFA stratified by potential modifiers.**

*Notes:*

HFA, height for age.

The adjusted models were adjusted for age, gender, low birth weight, being ill for the last two weeks, optimal feeding scores, residence, maternal education level, maternal height, maternal weight, mother suffering from anemia during pregnancy, antenatal visits, wealth status, drinking water source.

P value for difference: Z test was used to test for statistically significant difference in $\beta$ estimates across categories within subgroups. For example, in rural area vs urban area, we calculated: Z=$\frac{|\beta_{\mathrm{urban}}-\beta_{\mathrm{rural}}|}{\sqrt{\mathrm{se}_{\mathrm{urban}}^{2}+\mathrm{se}_{\mathrm{rural}}^{2}}}$.

**
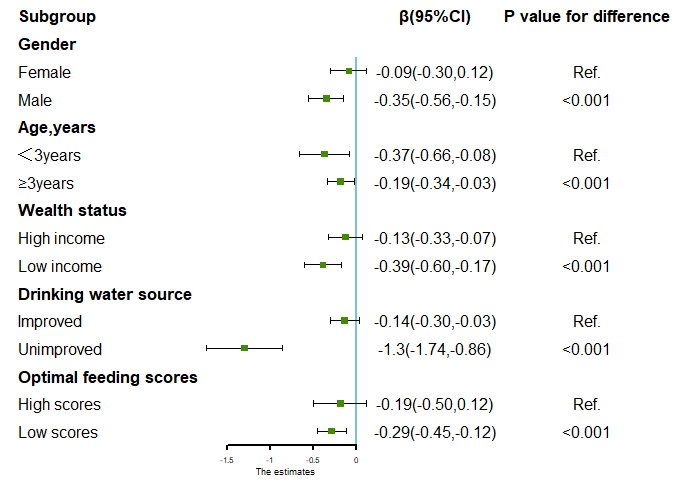
Figure S7 Association between altitude and Z-scores of WFA stratified by potential modifiers.**

*Notes:*

WFA, weight for age.

The adjusted models were adjusted for age, gender, low birth weight, being ill for the last two weeks, optimal feeding scores, residence, maternal education level, maternal height, maternal weight, mother suffering from anemia during pregnancy, antenatal visits, wealth status, drinking water source.

P value for difference: Z test was used to test for statistically significant difference in $\beta$ estimates across categories within subgroups. For example, in rural area vs urban area, we calculated: Z=$\frac{|\beta_{\mathrm{urban}}-\beta_{\mathrm{rural}}|}{\sqrt{\mathrm{se}_{\mathrm{urban}}^{2}+\mathrm{se}_{\mathrm{rural}}^{2}}}$.

**
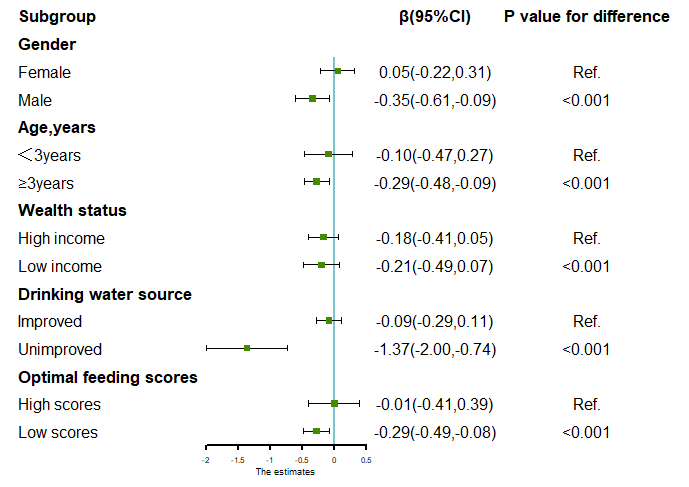
Figure S8 Association between altitude and Z-scores of WFH stratified by potential modifiers.**

*Notes:*

WFH, weight for height.

The adjusted models were adjusted for age, gender, low birth weight, being ill for the last two weeks, optimal feeding scores, residence, maternal education level, maternal height, maternal weight, mother suffering from anemia during pregnancy, antenatal visits, wealth status, drinking water source.

P value for difference: Z test was used to test for statistically significant difference in $\beta$ estimates across categories within subgroups. For example, in rural area vs urban area, we calculated: Z=$\frac{|\beta_{\mathrm{urban}}-\beta_{\mathrm{rural}}|}{\sqrt{\mathrm{se}_{\mathrm{urban}}^{2}+\mathrm{se}_{\mathrm{rural}}^{2}}}$.

**
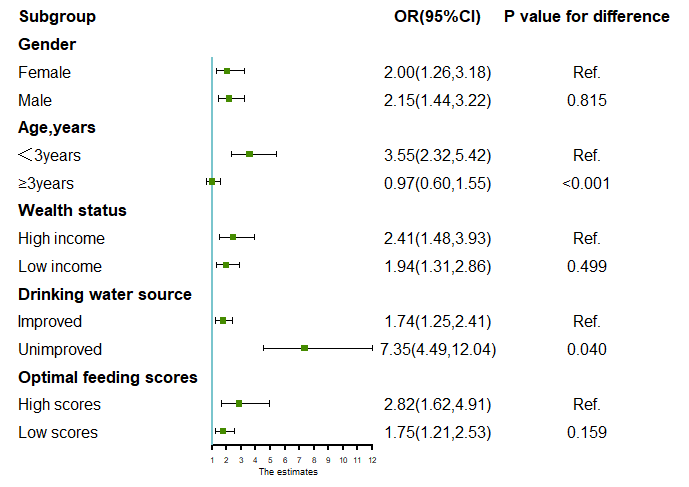
Figure S9 Association between altitude and stunting stratified by potential modifiers.**

*Notes:*

The adjusted models were adjusted for age, gender, low birth weight, being ill for the last two weeks, optimal feeding scores, residence, maternal education level, maternal height, maternal weight, mother suffering from anemia during pregnancy, antenatal visits, wealth status, drinking water source.

P value for difference: Z test was used to test for statistically significant difference in OR estimates across categories within subgroups. For example, in rural area vs urban area, we calculated: Z=$\frac{|\mathrm{OR}_{\mathrm{urban}}-\mathrm{OR}_{\mathrm{rural}}|}{\sqrt{\mathrm{se}_{\mathrm{urban}}^{2}+\mathrm{se}_{\mathrm{rural}}^{2}}}$.

**
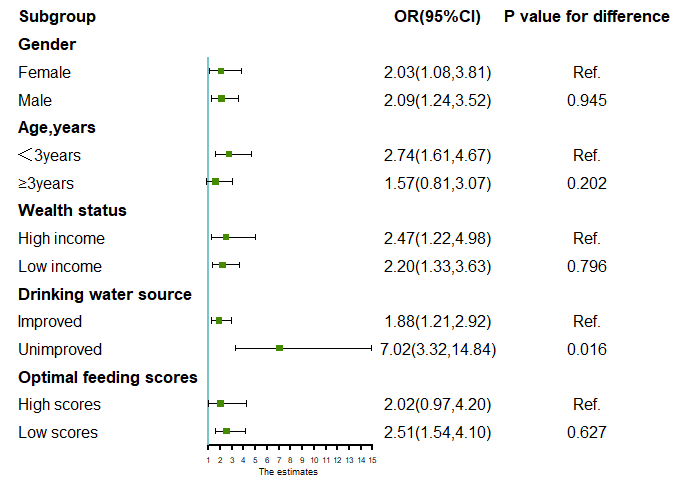
Figure S10 Association between altitude and underweight stratified by potential modifiers.**

*Notes:*

The adjusted models were adjusted for age, gender, low birth weight, being ill for the last two weeks, optimal feeding scores, residence, maternal education level, maternal height, maternal weight, mother suffering from anemia during pregnancy, antenatal visits, wealth status, drinking water source.

P value for difference: Z test was used to test for statistically significant difference in OR estimates across categories within subgroups. For example, in rural area vs urban area, we calculated: Z=$\frac{|\mathrm{OR}_{\mathrm{urban}}-\mathrm{OR}_{\mathrm{rural}}|}{\sqrt{\mathrm{se}_{\mathrm{urban}}^{2}+\mathrm{se}_{\mathrm{rural}}^{2}}}$.

**
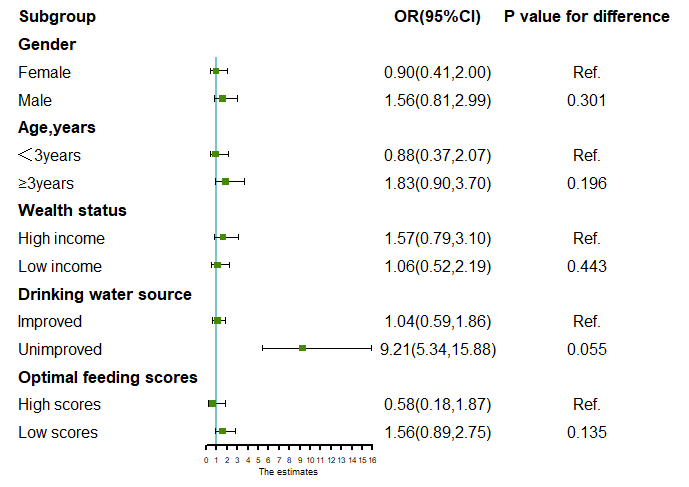
Figure S11 Association between altitude and wasting stratified by potential modifiers.**

*Notes:*

The adjusted models were adjusted for age, gender, low birth weight, being ill for the last two weeks, optimal feeding scores, residence, maternal education level, maternal height, maternal weight, mother suffering from anemia during pregnancy, antenatal visits, wealth status, drinking water source.

P value for difference: Z test was used to test for statistically significant difference in OR estimates across categories within subgroups. For example, in rural area vs urban area, we calculated: Z=$\frac{|\mathrm{OR}_{\mathrm{urban}}-\mathrm{OR}_{\mathrm{rural}}|}{\sqrt{\mathrm{se}_{\mathrm{urban}}^{2}+\mathrm{se}_{\mathrm{rural}}^{2}}}$.

**Table S1 Association of risk of malnutrition of children per 1000 m increments in altitude after adjusting for three preexisting diseases including asthma, anemia, and dental caries.**

| **Outcome** | **Altitude** | | |
| --- | --- | --- | --- |
|  | **Crude model^a^** | **Adjusted model 1^b^** | **Adjusted model 2^c^** |
|  | **β(95%CI)** | **β(95%CI)** | **β(95%CI)** |
| **Z-scores of HFA** | -0.39(-0.50, -0.27)^***^ | -0.38(-0.50, -0.27) ^***^ | -0.18 (-0.34, -0.02) ^*^ |
| **Z-scores of WFA** | -0.36(-0.47, -0.25) ^***^ | -0.35(-0.45, -0.24) ^***^ | -0.27(-0.43, -0.11) ^***^ |
| **Z-scores of WFH** | -0.20(-0.34, -0.06) ^**^ | -0.18(-0.32, -0.05) ^**^ | -0.26(-0.46, 0.06) ^*^ |
|  | **OR(95%CI)** | **OR(95%CI)** | **OR(95%CI)** |
| **Stunting**^d^ | 2.12(1.74, 2.59)^***^ | 2.20(1.79, 2.71) ^***^ | 1.83 (1.31, 2.56) ^***^ |
| **Underweight**^e^ | 1.98(1.47, 2.65) ^***^ | 1.94(1.45, 2.61) ^***^ | 2.13(1.35, 3.37) ^***^ |
| **Wasting**^f^ | 1.52(1.06, 2.19) ^**^ | 1.49(1.03, 2.14) ^**^ | 1.17(0.68, 2.02) |

*Notes:* HFA, height for age; WFA, Weight for age; WFH, weight for height.

**^a^Crude model:** no adjustment.

**^b^Adjusted model 1:** adjusted for age, gender, low birth weight, being ill for the last two weeks.

**^c^Adjusted model 2:** adjusted for age, gender, low birth weight, being ill for the last two weeks, optimal feeding scores, residence, maternal education level, Maternal height, maternal weight, mother suffering from anemia during pregnancy, antenatal visits, wealth status, drinking water source.

^d^Stunting: Z scores of HFA<-2.

^e^Underweight: Z scores of WFA<-2.

^f^Underweight: Z scores of WFA<-2.

***, P value <0.001;**, P value is between 0.001 and 0.01; *, P value is between 0.01 and 0.05.

**
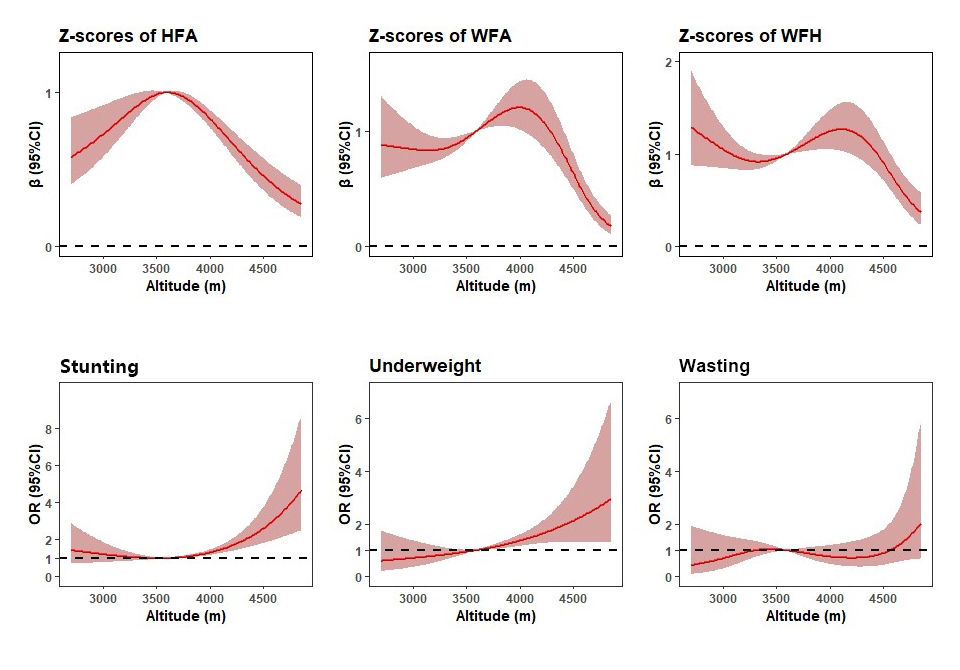
Figure S12 Curve associations between malnutrition indicators and altitude exposure.**

*Notes:*

HFA, height for age; WFA, Weight for age; WFH, weight for height.

The adjusted models were adjusted for age, gender, low birth weight, being ill for the last two weeks, optimal feeding scores, residence, maternal education level, Maternal height, maternal weight, mother suffering from anemia during pregnancy, antenatal visits, wealth status, drinking water source.
